# No signs of automatic perspective-taking or its modulation by joint attention in toddlers using an object retrieval task

**DOI:** 10.1098/rsos.220347

**Published:** 2022-08-03

**Authors:** Qianhui Ni, Bella Fascendini, Jake Shoyer, Henrike Moll

**Affiliations:** ^1^ Department of Psychology, University of Southern California, 3620 S. McClintock Avenue, Los Angeles, CA 90089-1061, USA; ^2^ Department of Psychology, Stanford University, 450 Serra Mall, Stanford, CA 94305, USA

**Keywords:** perspective-taking, social cognition, mind-reading, joint attention

## Abstract

It is currently debated whether simple forms of social perspective-taking that are in place by late infancy are performed automatically. We conducted two experiments (*N* = 124) to test whether 3-year-olds show automatic perspective-taking during object searches, and whether automatic perspective-taking is facilitated by joint attention. Children were asked to retrieve an object immediately after it was moved from one (L1) to another (L2) location within a container, e.g. a sandbox. In Experiment 1, a between-subjects design was used, with children being randomly assigned to one of three experimental conditions: one in which child and other jointly attended to the object in L1 (joint attention condition); one in which the other was present but unengaged with the child when the object was placed in L1 (other present condition) and a baseline condition in which only the child was present (no other condition). Automatic perspective-taking should manifest in biased searches toward L1 in the other present and joint attention conditions, but not in the no other condition. No automatic perspective-taking was observed in either experiment, regardless of whether the other person left and remained absent (Experiment 1) or returned after the object was relocated (Experiment 2). The findings contribute to a growing body of empirical data that questions the existence of automatic perspective-taking.

## 1. Introduction

One of the hallmarks of human cognition is the ability to view or imagine situations from perspectives other than one's own. The capacity to take others' perspectives pervades human interaction and is key for healthy social development [1]. Communication and cooperation are contexts in which having a sense of the other's perspective is especially important in order to coordinate one's activities with those of one's partner (e.g. [2,3]).

Numerous studies in cognitive development and developmental pragmatics have established that simple forms of perspective-taking are in place by late infancy or toddlerhood. For example, 2-year-olds grasp when others are not able to see an object they themselves can see—a capacity known as level 1 visual perspective-taking ([4,5]; see also [6], for positive findings with younger infants using looking-time measures). Even before children can engage in visual perspective-taking, they engage in what might be called ‘experiential perspective-taking’: they know what others have and have not witnessed and thus are and are not familiar or acquainted with from prior experience (see [7], for a review). Action measures have revealed that 1-year-olds can track which objects another person has and has not interacted with [8,9], and looking-time studies suggest an even earlier ability to track others' experiences (e.g. [10,11]).

It has been proposed that these basic forms of visual and experiential perspective-taking are not only early emerging but effortless and even automatic. The two-systems account of mind-reading argues that humans and certain other animals are innately endowed with a mind-reading system (System 1) that automatically tracks what objects and events others encountered and where [12,13]. This system's purpose is to facilitate social coordination. For example, knowing what is and is not within another's line of sight allows subordinate apes to avoid clashing with higher-ranked individuals [14], and tracking others' object engagements allows toddlers to secure reference and communicate effectively (e.g. [15]). If perspective-taking of this kind is automatic, as has been suggested, then interferences are bound to occur when others’ perspectives conflict with one's own but must be ignored. One's own individual action and judgement would be slowed and error-prone when another's perspective differs, even when the other's perspective is entirely irrelevant to what one is doing.

Several sets of findings suggest that such interference effects exist. Samson *et al*. [16] had adult participants report the number of dots in a visual scene. Participants responded slower and with more errors when an avatar embedded in the scene saw only some of the dots in the scene the participant saw (incongruent perspective) than when the avatar saw the same number of dots (congruent perspective). Surtees *et al*. [17,18] replicated the findings with both adults and school children, suggesting that humans, across developmental stages, represent others' perspectives even when it impairs performance (see also [19]). The opposite effect—*enhanced* performance due to automatic perspective-taking—has also been reported, with adults detecting low-contrast patterns faster in the co-presence of an avatar ([20], see also [21]). In another study, participants inhibited distractors not only of their own but also of others’ manual tasks, indicating that they automatically shifted to allocentric frames of reference [22]. Similarly, Sebanz and co-workers [23,24] report that humans co-represent each others' tasks, even when ignoring the other's task would be beneficial for their own individual task execution.

Developmentalists argue that infants are particularly prone to experiencing interference from others' perspectives because they are still in the process of forming a representation of themselves [25,26]. Supporting this idea, looking-time data suggest that infants process events they witness in others’ company more deeply than events they experience solo, with the effect that co-witnessed, but outdated, information is prioritized over privileged, but newer, information [27].

Despite these reports, the existence of automatic perspective-taking remains contested. Several recent studies using the dot-perspective paradigm found no signs of automatic perspective-taking ([28–30]; see also an ongoing meta-analysis [31]). In one of these studies, performance *was* lower when another's perspective was incongruent compared with when it was congruent with that of the participant. However, the same congruency effect occurred when the agent was replaced by an asymmetrically shaped artefact (a desk-fan), suggesting that attentional cueing accounts for the effect [29]. The proposal that young children prioritize others' perspectives over their own has also failed to receive support from recent studies [28,32]. For example, a study with 3- to 6-year-olds showed that children only displayed sensitivity to others’ viewpoints when asked to think about the other's perspective, but not otherwise [32]. This conflicts with the idea of automaticity, which implies a general bias toward others' perspectives, without any explicit request to think about the other's viewpoint.

To shed light on the question of whether, and, if so, under what social conditions, automatic perspective-taking might be detected, we investigated whether young children's object retrievals are biased to another's outdated perspective. In two experiments, we used an online version of the sandbox task [33,34], in which children first see an object placed in one location (L1) and then moved to another (L2) within a sandbox (or planter box, etc.). Three-year-olds were chosen because they are widely agreed to have the experiential perspective-taking skills handled by System 1 and are old enough to follow digital instructions and perform touch-screen interactions [35].

In the first experiment, we varied whether another person besides the child was absent (no other condition) or present when the object was placed in L1 (with only the child watching the object's relocation to L1, regardless of condition). We furthermore manipulated whether the person was merely present (other present condition) or jointly attending to the object with the child (joint attention condition). The guiding question in this experiment was thus whether joint attention modulates automatic perspective-taking. The next paragraph lays out reasons to assume that joint attention promotes automatic perspective-taking. In response to negative findings in the first experiment, we sought to increase the chances of detecting potential automatic perspective-taking by having the person return to the scene after the object's relocation and prior to the child's object retrieval. This was done to facilitate children's retrieval of their record of what the other person had last witnessed, thus enhancing the likelihood of searches biased toward that person's perspective on the object's location.

There are reasons to assume that joint attention promotes automatic perspective-taking. In joint attention, infants represent others' experiences better than outside of joint attention, as shown by findings indicating that infants can distinguish between what is new versus old for others only if the ‘old’ objects were jointly experienced [8,36]. Brain studies found that the negative component of event-related potentials increases in amplitude—a neural indicator of attention—when infant and other jointly attend to an object but not when they attend to it separately [37]. In another study, infants looked longer at an object they had jointly attended to with someone compared with a new object—a difference that was absent when the first object was co-witnessed, but not jointly attended to by child and other ([38]; see also [39]). In sum, *jointly* witnessed objects and events are encoded more deeply than co-witnessed, but not jointly witnessed ones. Greater interference by an irrelevant perspective with a child's performance when that perspective represents a prior ‘we perspective’, i.e. a perspective that child and other shared, compared with a perspective that child and other each held separately. We investigated this in Experiment 1.

## 2. Experiment 1

The experiment was approved by the University's Institutional Review Board and pre-registered at https://doi.org/10.17605/OSF.IO/HBQY6 at the Open Science Framework. In three conditions, 3-year-olds were asked to retrieve an object that was relocated from one (L1) to another (L2) location inside a long skinny container (e.g. a sandbox). The dependent measure was the degree to which children's object searches were biased toward L1. We manipulated whether the object in L1 was co-witnessed by another person and, if so, whether the child and other person were in joint attention. If children take the other's perspective automatically, their searches should be biased toward L1 in the other present and/or joint attention condition, but not, or less so, in the no other condition. And if joint attention enhances such perspective-taking, then children's search bias toward L1 should be greater in the joint attention than the other present condition.

### 2.1. Methods

#### 2.1.1. Participants

An *a priori* power analysis with alpha set at 0.05 and power of 0.80 (G*Power v. 3.1, *ANOVA*: repeated measures between factors) yielded that a sample size of 72 children would be needed to detect a moderate effect of 0.3. Our final sample included 72 (36 female) 3-year-olds (mean = 39.52 months; range = 32.40–45.57 months); with a mean age of 38.02 months in the joint attention condition, 39.53 months in the other present and 41.01 months in the no other condition. Three further children (one from each condition) were excluded due to uncooperativeness. The racial/ethnic composition of the final sample was 11% multi-racial, 7% African American, 17% Asian, 54% White, 1% American Indian or Alaska Native, and 10% ‘other’; 14% were Hispanic. The sample represented diverse socio-economic backgrounds, with reported household incomes ranging from less than $20 000 to greater than $120 000. Participants were recruited from local preschools, social media and websites offering research participation for children.

#### 2.1.2. Material

Children used a computer (tablet, laptop or desktop) with touch-screen function and a minimum screen size of 9.7 inches. In the *familiarization phase*, two pairs of a container and matching object were shown against white background. These were a blue bucket (15% of screen width and 73% of screen height) with a multi-coloured ball (10% of screen width/height), and a yellow basket (22% of screen width and 96% of screen height) with a red apple (10% of screen width and 54% of screen height).

**Figure 1.**
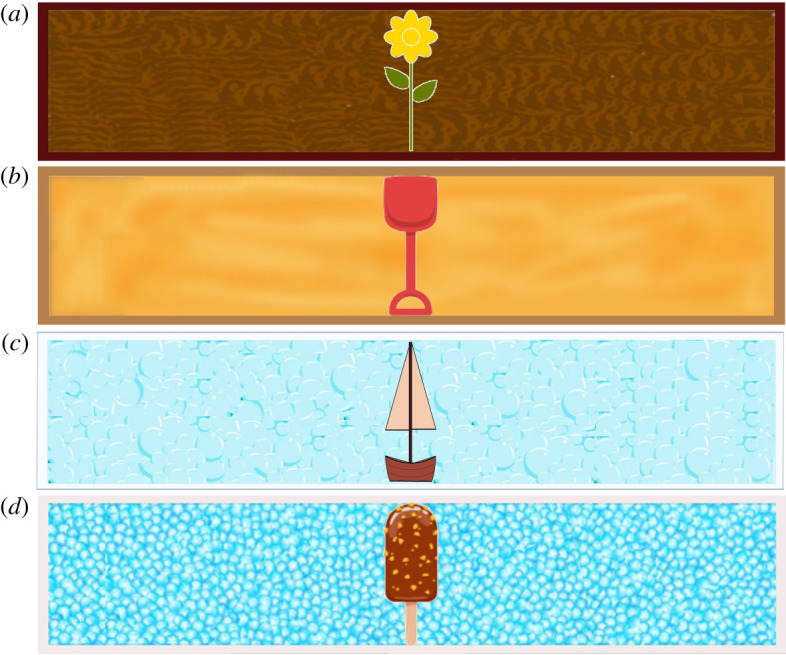
Objects and containers used in the object retrieval task. Four pairs of objects and corresponding containers were used: (*a*) a flower in a planter box, (*b*) a shovel in a sandbox, (*c*) a boat in a bathtub and (*d*) an ice-cream bar in a freezer.

In the *test phase*, a long, narrow container (planter box, sandbox, bathtub and freezer) filled the entire screen. It was presented with a matching object (flower, shovel, boat and ice-cream bar), which filled the container in height and consumed about 7% of its width, as shown in figure 1.

#### 2.1.3. Design

A between-subject design was used, with 24 children (12 female) randomly assigned to one of three conditions. All conditions included a *familiarization phase* (consisting of two practice trials digging an object out of a container by touching the computer screen), followed by a *test phase* consisting of four object retrieval trials. There were four relocation patterns, with the object being moved either from side to centre (L → C, R → C) or from centre to side (left or right; C → L, C → R). Each child received each relocation pattern once, with order of object–container pairings and relocation order varying between children.

#### 2.1.4. Procedure

The experiment involved three parties in a Zoom session: the child with her parent, a female or male experimenter (E), and a background assistant who controlled the animations without being visible or audible throughout the experiment. The parent had given consent and provided demographic information prior to the session. The parent ensured that the child sat about 45 cm in front of the computer, that E's video was pinned, and that screen touches were recorded by Zoom's annotation feature.

*Familiarization Phase**.*** This phase was identical across conditions and served to demonstrate to children that they could retrieve objects from a container by touching the screen. E asked the child to dig out a ball (and next, an apple) immediately after it was placed in a bucket (or basket).

**Figure 2.**
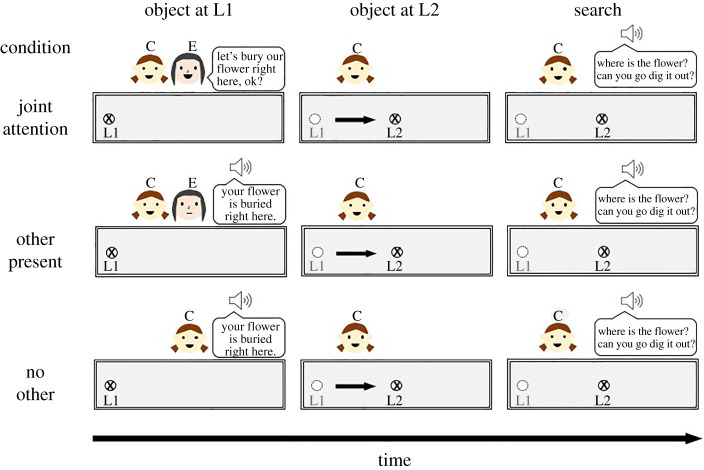
Experimental procedure by condition. The object is dug into L1 either while child (C) and E are present (other present condition) and jointly attending (joint attention condition), or while only the child is present (no other condition). Next, the object is moved to L2 in only the child's presence. Finally, a synthetic voice prompts the child to retrieve the object. Note: the child's and E's spatial positions in this figure were chosen based on ease of depiction. They do not reflect actual spatial positions in the online experiment, in which child and E were visible for one another via their respective cameras.

*Test Phase.* This phase varied by condition. Figure 2 shows the key procedural steps and differences between conditions. In the **joint attention condition**, E said to the child: ‘Look, here is a flower – do you like it?’ After the child answered, E stated, e.g. ‘I like this flower, too. This flower is our flower now’. E then announced that the two would dig their flower into the planter box together, stating ‘Let's bury our flower in the planter box’. The planter box then appeared on the screen. E contemplated ‘Hm, where should we bury our flower?’ as the flower hovered over the box. E exclaimed ‘Let's bury our flower right here, ok?’ The flower was then dug into L1. E took leave (I have to go now, bye!) and then turned his/her video off. After E left, the object was relocated from L1 to L2. Next, a synthetic voice asked the child ‘Where is the flower? Can you go dig it out?’ The request was repeated up to three times. After the child responded by touching the screen, the procedure was repeated with the next object–container pair.

In the **other present condition**, everything was as above with the difference that E did not interact with the child. After the object appeared, a synthetic voice stated: ‘Look, there is [*E's name*]. He/she is watching! This flower looks nice. This flower is your flower now. Your flower is going to be buried in the planter box’. The object hovered over the box and the synthetic voice announced ‘Your flower is buried right here’. The object was then placed in L1 while child and E were co-present but not interacting. After the object was dug in, the procedure continued as in the joint attention condition. In the **no other condition**, E left after the familiarization phase and did not return. The rest of the procedure was as described for the other present condition.

The session lasted approximately 15 min and was concluded with the parent's receipt of a $15 gift card.

#### 2.1.5. Scoring, reliability and analyses

The first author took screenshots from the video recordings of children's screen touches in every test phase. An independent rater, unaware of condition, placed a scale from 0 to 100 over each screenshot and determined a ‘touch location score’ by identifying the number on the scale where the screen was touched. If the screenshot indicated no location where the screen was touched (which occurred in 9 out of 288 trials), the rater coded ‘no response’. A second rater, also unaware of condition, applied the same scoring procedure for a randomly selected 25% of the sample. Inter-rater reliability was excellent, with 93% agreement (*Kappa* = 0.93). Disagreements were resolved by discussion.

The object's actual location, L2, had a score of 4 (left), 50 (centre) or 96 (right) on the scale. A bias score was calculated for each trial by subtracting the touch location score from L2. Bias scores could range from −96 to +96, with *positive* scores indicating a bias *toward* L1 and *negative* scores indicating a bias *away from* L1. A bias score of 0 meant that the child accurately retrieved the object from L2.

### 2.2. Results

A repeated-measures analysis of variance (ANOVA) found no effects of age, gender, race or order of container–object pairs on children's bias scores, *p*s > 0.20. The mean bias scores for each condition were +8.64 (s.d. = 20.0) for the joint attention, +8.56 (s.d. = 21.1) for the other present and +9.28 (s.d. = 20.1) for the no other condition. To determine whether children's searches were biased, we ran one-sample *t*-tests with Bonferroni-corrected *p*-values to measure whether mean scores in each condition differed from 0 (L2). The tests showed that children's searches were significantly biased away from L2 and toward L1 in all conditions (joint attention condition: *t*_90_ = 4.12, *p* < 0.001, Cohen's *d* = 0.43; other present condition: *t*_92_ = 3.91, *p* < 0.001, Cohen's *d* = 0.41; no other condition: *t*_94_ = 4.50, *p* < 0.001, Cohen's *d* = 0.46).

**Figure 3.**
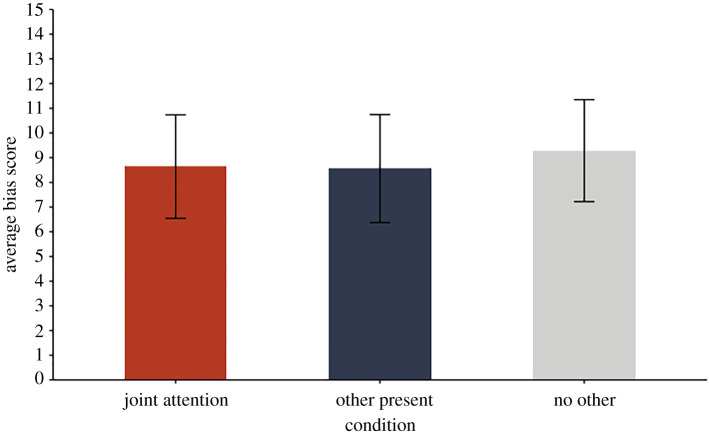
Average bias scores as a function of condition. Error bars represent standard errors.

To determine whether biases differed between conditions, we ran a repeated-measure ANOVA with condition as an independent variable and bias score as a dependent variable. There was no condition effect, *F*_2,276_ = 0.04, *p* = 0.97, *η*^2^ < 0.001 (figure 3), indicating that children's search bias was identical independent of whether a person was jointly attending with them, present or absent when the object was placed in its outdated location (L1). The cause of this bias thus cannot be automatic perspective-taking.

To explore the source of the bias, we compared different relocation patterns. An ANOVA with relocation pattern (C → L, C → R, L → C, R → C) as an independent variable and bias score as a dependent variable showed a strong effect of relocation pattern, *F*_3,275_ = 14.09, *p* < 0.001, *η*^2^ = 0.13. *Post hoc* tests using Tukey's HSD showed that the bias was caused by trials in which the object was moved from the centre to the side, *p*s < 0.001, not by those in which it was moved from side to centre, *p*s = 0.99. One-sample *t*-tests (with Bonferroni-corrected *p*-values) found that children's searches significantly diverged from 0 (L2) only when the object was moved to the side (mean = 16.26), *t*_138_ = 8.24, *p* < 0.001, Cohen's *d* = 1.40, not when it was moved to the centre (mean = 1.46), *t*_139_ = 1.29, *p* = 0.10, Cohen's *d* = 0.22, indicating that children tended to search for peripheral objects near the centre.

To look for possibly undetected signs of automatic perspective-taking in trials unaffected by the spatial bias, we reran the ANOVA with condition as an independent variable and bias score as a dependent variable with only those trials in which the object was relocated to the centre (trials which showed no spatial bias). As before, no effect of condition and thus no evidence of automatic perspective-taking was found, *F*_2,137_ = 0.01, *p* = 0.99.

### 2.3. Discussion

This experiment produced no signs of automatic perspective-taking. Children's object retrievals were not biased toward another's outdated information about where the objects were located. Along with other findings [28,29,40–44], the results question the idea that children involuntarily take others' perspectives.

A non-social spatial bias was detected for half of the data. Biases toward the centre of the container were observed whenever the object's final location, L2, was at the periphery. This demonstrates the importance of looking out for spatial and other low-level biases as potential explanans of performance impairments. The possibility of such biases must be carefully considered when interpreting children's object searches, e.g. in the context of continuous false-belief tasks [33,34]. Proper controls either in the form of perspective-congruent (versus incongruent) or non-social (versus social) conditions (the no other condition in this experiment) are key for preventing such biases from being misinterpreted as perspective-taking or false-belief sensitivity.

In the next experiment, we sought for signs of automatic perspective-taking in 3-year-olds' object searches under altered conditions. First, we eliminated the spatial bias detected in this experiment by using only side-to-centre relocations, in which this bias was absent. Second, we changed the procedure such that the person who failed to witness the object's relocation to L2 returned before the child's response. Why might the person's return to the scene impact automatic perspective-taking?

Unlike in visual perspective-taking tasks, in which the perspective difference between child and other is established in the moment the child responds, in ‘experiential perspective-taking’ tasks (like the sandbox task), the perspective difference was established in earlier, past, experiences and so the other agent could—depending on the specific task—either remain absent or return to the scene as the child responds. What is noteworthy is that in studies reporting that infants are sensitive to others' perspectives, the other was brought back to the scene as the infant responded. In Kovács *et al*.'s [10] study, the Smurf who failed to witness a ball rolling away from behind a barrier, returned just before the ball's absence was revealed to the infant. Similarly, in anticipatory-looking and other measures of sensitivity to others' beliefs, the agent either returns or her return is announced to prompt the child's response [45,46]. The agent's return might be crucially important, as is suggested by Perner & Roessler's [47,48] idea of ‘experiential records'. In this account, toddlers maintain records of what others experience, and these records are activated in the others' presence. As the authors state: ‘Infants keep track of what agents perceive (experiential record); in particular, the state of the world last seen by the agent. Focus on the agent activates this record and induces them to construe the agent's actions or to anticipate future actions on the basis of this record’ [48, p. 524]. To enhance children's retrieval of the experiential record and thereby increase the likelihood of perspective-taking, in the next experiment, we had the other person return to the container just before children retrieved the object.

## 3. Experiment 2

The experiment was approved by the University's Institutional Review Board and was pre-registered at the Open Science Framework (https://doi.org/10.17605/OSF.IO/WS72K). The same object retrieval paradigm as in Experiment 1 was used. Children were randomly assigned to the joint attention or the no other condition. The other present condition from Experiment 1 was not rerun because children's object retrievals were found not to differ between the two sharply opposed conditions.

### 3.1. Method

#### 3.1.1. Participants

Participants were 52 3-year-olds (26 females) with a mean age of 37.53 months, ranging from 32.97 months to 43.43 months. Another two children were tested but excluded due to uncooperativeness. This sample size was decided based on a prior power analysis with alpha set at 0.05 and power of 80% in the aim of detecting a moderate effect size of 0.30.

Participants were recruited from social media, friends and families. The racial/ethnic composition of the sample was 10% with African American, 13% Asian, 52% White, 12% multi-racial, 2% Native Hawaiian or Native Pacific Islander, 4% other, and 8% not indicated. Socio-economic status as estimated by household income was diverse (with incomes ranging from less than $20 000 to greater than $120 000).

#### 3.1.2. Material and design

The same materials as in Experiment 1 were used. Two additional object-container pairs were used: a (rice-filled) lunchbox with a carrot and a (foam-filled) cardboard box with a toy giraffe.

An equal number of children (*n* = 26, 13 females in each) was randomly assigned to the joint attention and the no other condition. The familiarization phase was the same as in Experiment 1. The test phase included six, not four, trials. Objects were relocated from left to centre on three trials and from right to centre on the remaining three trials. The order of container–object pairs and relocation order were counterbalanced.

#### 3.1.3. Procedure

The procedure of the no other condition was identical to that in Experiment 1. The procedure of the joint attention condition was also the same with the exception that E returned to the scene just before the child was asked to dig out the object. After the object was moved from L1 to L2, E turned her video back on and said ‘Hi, I'm back’.

#### 3.1.4. Scoring and reliability

The same scoring and reliability procedures as in Experiment 1 were applied. Inter-rater reliability was excellent (*Kappa* = 0.87). Disagreements were resolved by discussion.

Children's bias scores were calculated in the same way as in Experiment 1. The object's final location (L2) had a score of 50, with bias scores potentially ranging from −50 to +50.

### 3.2. Results

**Figure 4.**
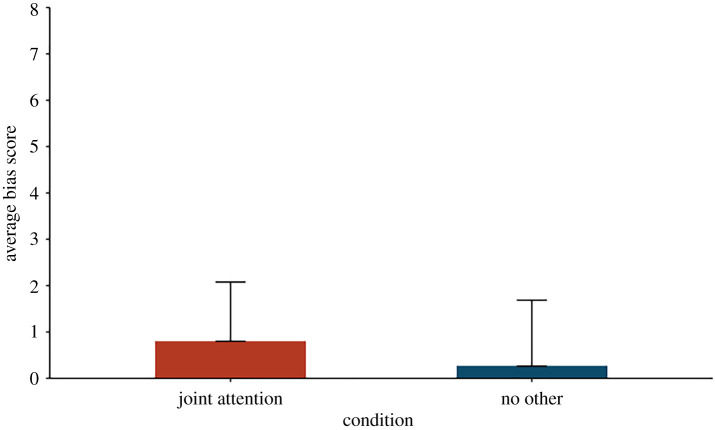
Average bias scores as a function of condition. Error bars represent standard errors.

An ANOVA with repeated measures found no effects of gender, age, race or order of container–object pairs, *p*s > 0.18. Mean bias scores were +0.80 (s.d. = 15.70) and +0.26 (s.d. = 17.55) for the joint attention and the no other condition, respectively. A repeated-measures ANOVA with condition as an independent variable and bias score as a dependent variable showed that children's search locations were the same for the no other and joint attention conditions, *F*_1,301_ = 0.08, *p* = 0.78, (figure 4). Furthermore, one-sample *t*-tests comparing children's searches in each condition against 0 (L2) were not significant, indicating that children's searches were accurate, rather than biased, in both conditions, *p*s > 0.27.

### 3.3. Discussion

This experiment, like the previous one, revealed no traces of automatic perspective-taking. Where children looked for an object did not differ depending on whether they were alone or in joint attention with another person when the object was placed in a location from which it was later removed. Changing the procedure by having the person with the outdated information of the object's location return to the scene before children retrieved the object did not lead to automatic perspective-taking. Bringing the person back to the scene should have activated children's access to their record of their and the other's prior engagement with the object in its outdated location [48], thereby increasing the chances of potentially existing automaticity in perspective-taking to be found. Yet, even under these circumstances that are most conducive to eliciting automatic perspective-taking, it was still not observed.

## 4. General discussion

In this study, we used an online version of the sandbox task [34,49] to investigate automatic perspective-taking and its potential modulation by social factors in toddlers. In two experiments, 3-year-olds retrieved an object from a container after it was moved from one (L1) to another (L2) place in a container. We tested whether children's retrievals were biased toward L1, the object's outdated location, depending on whether another person was jointly attending, present but unengaged with the child, or absent when the object was placed in L1. Experiment 1 found no signs of automatic perspective-taking, as would be evidenced by a bias toward L1 when the other was present and/or in joint attention with them (other present condition and joint attention conditions) but not when participants were alone (no other condition). In Experiment 2, a comparison of the joint attention and no other condition again yielded no effect of another's presence and joint attention on children's searches. In sum, no experimental evidence in support of the claim that perspective-taking is involuntary or automatic was found.

The null findings are consistent with other reports of an absence of automatic perspective-taking, both in adults [29,31,40–44,50] and children [28,32]. Some of these other studies also used the sandbox paradigm [32,50]. These results conflict with reports that children and adults alike tend to involuntarily take others' point of view, as seen by impacts on their individual performance when another's, irrelevant, point of view diverges from theirs [16,17,51,52]. Similarly, it has been reported that humans are inclined to represent the tasks and goals of those around them, even when such co-representation distracts them from their own task [19,24,53].

The negative results challenge the two-systems account of human mind-reading, which argues that simple forms of perspective-taking, handled by the evolutionarily ancient System 1, are not just effortless—e.g. they hold up under cognitive load [18]—but also automatic. The general idea is that the effortlessness of System 1, although it enables smooth and effective interactions with conspecifics, comes at a cost. The cost is that System 1 cannot deliberately be turned on or off. It operates regardless of volition, with the effect that those who have System 1 *cannot help* but process a situation from another's point of view [12,54], resulting in undesired performance impairments due to interferences from others' incongruent perspectives.

One might try to argue that basic, level-1-type, perspective-taking is automatic, as the two-systems account claims, but that tracking another's perspective in the sandbox task requires higher, level-2, perspective-taking. The task is modelled after the location-change false-belief task, which, in its standard form, is known to be a level-2 problem that children younger than age 4 consistently fail [55]. Several studies have confirmed that level-2 problems are beyond System 1's scope and thus *not* subject to automatic perspective-taking [40,56,57]. Yet, this explanation fails because an agent's failure to witness the relocation of a previously encountered object (leading to the agent's ‘registering’ the object in its outdated location) constitutes a paradigm case for System 1 (see [12, pp. 962–963]; [13, pp. 1–12]). The two-system account emphasizes that tracking agent-object relations across time subserves social coordination in animals including birds, primates and humans thanks to their shared possession of this cost-efficient but rigid system.

An alternative possibility is that automatic perspective-taking is present in infancy but disappears as infants turn into toddlers—hence our negative finding with 3-year-olds. Something like this is suggested by Southgate [26], who claims that altercentrism is strong in early infancy but wanes as infants develop a firm representation of themselves, as reflected by success in mirror self-recognition and other self-awareness tests [58–60]. The problem with this account is that these tests indicate a *third-personal* understanding of oneself [61,62]—the self as seen from an outside perspective—which is a different issue altogether to the fundamental fact that any sentient being, whether it has self-awareness or not, views the world from a particular visuo-spatial position. It is this latter fact about our perspectival access to the world that matters for interpersonal perspective-taking. Automatic perspective-taking, if it exists, should thus persist beyond infancy, especially if its purpose is, as the two-systems view argues, ease of social interaction (which improves as infants mature).

Our own suggestion is to rethink the notion of automatic perspective-taking by taking a systematic look at the circumstances under which it occurs. One issue is that interferences from other sources might be misinterpreted as perspective-taking. You might be familiar with the phenomenon of advancing from a stop at a red light because cars next to you start moving or because a green signal belonging to a different lane catches your eye. In situations like these, our actions are affected by conflicting information, but not because we take a particular person's perspective. Something like this could cause task co-representation effects: hearing an instruction, although not meant for you, makes you inclined to act on that instruction—regardless of whether another agent receives or acts on that instruction. Separating automatic perspective-taking from these influences requires tight controls, in which participants perceive to-be-ignored signals and instructions in the absence of other agents.

Reductive explanations can also be given for dot-perspective and pattern detection tasks, in which the co-attender and stimuli are all within the same visual frame. In these visuo-spatial tasks, it is likely that the co-attender's physical presence and orientation in space biases participants to focus on certain parts of the screen, without taking the co-attender's perspective [29]. In sum, interpretive caution is required to avoid mistaking attentional cueing and other biases for automatic perspective-taking. Apart from these theoretical considerations, an empirical problem for the claim of automaticity in perspective-taking is that successful laboratory-independent replications are scarce, and negative findings like the ones reported here are accumulating [28,29,42,44].

Despite the doubt regarding automatic perspective-taking, we are sympathetic to the general idea that human children are prone to aligning their view with that of others and adopting their viewpoints. We take infants' proclivity to want to share experiences in joint attention [63,64], to imitate others with the goal to do what the other does [65,66], to build common ground by forming ‘conceptual pacts’ in conversation [67,68] and, more generally, to adopt others' stance toward the world (see [69]) to be a fundamental fact about humans that is at the basis of their social-cognitive development. In some cases, children might be said to go overboard in their adoption of others' perspectives, such as when they imitate ineffective action steps or deny the ability to see someone who cannot see them (presumably because they make mutual visual attention a condition for seeing a person; see [70,71]). But despite the strong inclination to consider others' viewpoints, there is no solid evidence to suggest that perspective-taking occurs involuntarily or automatically.

Another null finding that requires discussion is the absence of a joint attention effect. In neither experiment did joint attention lead children to subsequently adopt their co-attender's perspective. One might take this result to conflict with experiments showing the importance of joint attention for infants' understanding of reference [8,36]. We do not think, however, that there is a conflict, and here is why. What the reference resolution studies suggest is that joint attention is a *sine qua non* for infants to recognize others’ experiences: infants need to actively participate in others' experiences (as a partner) to be able to track others’ experiences and resolve reference based on these experiences. Joint attention, it seems, facilitates one's access to other minds in infancy. The experiments reported here tested a different idea, namely that children are *involuntarily biased* to another's perspective after having shared that perspective with her, leading to individual performance errors. The fact that this idea did not hold up does not question the importance of joint attention for the development of an understanding of other minds in infancy; it only questions the automatic nature of perspective-shifting.

One might doubt the generalizability of our findings to real-world situations and to other age groups. A worry might be that the online delivery of the experiments came with low ecological validity. In defence of the validity of online research, it has been shown that the COVID-19 pandemic has turned video chats into a frequent means of communication for young children [72]. A study comparing various social-cognitive skills (including competence judgements, false-belief understanding, etc.) of infants and young children in online versus in-person settings found no significant performance differences across the two settings [73]. Although these are reasons in favour of the current study's validity, we agree that in-person replications remain crucially important. Especially the success of an online operationalization of joint attention can be questioned, since joint attention in its paradigmatic form involves two (or more) agents experiencing some object together in a shared physical space. Such joint perceptual experiences can probably not be achieved, but only simulated, in an online format.

The second concern we mentioned pertains to the generalization of our findings to other ages. The two-system account states that automatic perspective-taking pervades the lifespan, because humans are said to be vulnerable to interference from alternative viewpoints as soon as they have the capacity for level-1 perspective-taking ([17]). We chose the age of 3 because level-1 perspective-taking is firmly in place and measurable with multiple techniques by this age [6,74]. Thus, at least according to the theory, age 3 is a valid testing ground for automaticity in perspective-taking, and its absence at this age is a problem for the automaticity claim more generally. Nonetheless, future work with multiple ages can help to determine potential age differences in one's tendency to be influenced by others' perspectives.

In conclusion, the current study does not suggest that basic, level-1-type, perspective-taking is automatic in the way the two-systems account argues [12,13]. Although our study does not prove that automatic perspective-taking does not exist, it offers reasons to doubt that automatic perspective-taking is a general feature of mind-reading. Specifically, the study found that young children's performance remained unbiased and immune to automatic perspective-taking, even in the context of joint attention and when the other agent was at the scene when children responded. Further research is required to determine whether there are conditions under which perspective-taking might be said to occur automatically, and, if so, what kinds of behaviour—e.g. attentional allocation, action or judgement—it tends to impact.

## Data Availability

The datasets supporting this article have been uploaded as part of the electronic supplementary material [75].
